# *Ppm1*-Encoded Polyprenyl Monophosphomannose Synthase Activity Is Essential for Lipoglycan Synthesis and Survival in Mycobacteria

**DOI:** 10.1371/journal.pone.0048211

**Published:** 2012-10-31

**Authors:** Amrita K. Rana, Albel Singh, Sudagar S. Gurcha, Liam R. Cox, Apoorva Bhatt, Gurdyal S. Besra

**Affiliations:** 1 School of Biosciences, University of Birmingham, Edgbaston, Birmingham, United Kingdom; 2 School of Chemistry, University of Birmingham, Edgbaston, Birmingham, United Kingdom; University of Delhi, India

## Abstract

The biosynthesis of mycobacterial mannose-containing lipoglycans, such as lipomannan (LM) and the immunomodulator lipoarabinomanan (LAM), is carried out by the GT-C superfamily of glycosyltransferases that require polyprenylphosphate-based mannose (PPM) as a sugar donor. The essentiality of lipoglycan synthesis for growth makes the glycosyltransferase that synthesizes PPM, a potential drug target in *Mycobacterium tuberculosis*, the causative agent of tuberculosis. In *M. tuberculosis*, PPM has been shown to be synthesized by Ppm1 in enzymatic assays. However, genetic evidence for its essentiality and *in vivo* role in LM/LAM and PPM biosynthesis is lacking. In this study, we demonstrate that *MSMEG3859*, a *Mycobacterium smegmatis* gene encoding the homologue of the catalytic domain of *M. tuberculosis* Ppm1, is essential for survival. Depletion of *MSMEG3859* in a conditional mutant of *M. smegmatis* resulted in the loss of higher order phosphatidyl-*myo*-inositol mannosides (PIMs) and lipomannan. We were also able to demonstrate that two other *M. tuberculosis* genes encoding glycosyltransferases that either had been shown to possess PPM synthase activity (*Rv3779*), or were involved in synthesizing similar polyprenol-linked donors (*ppgS*), were unable to compensate for the loss of MSMEG3859 in the conditional mutant.

## Introduction

Tuberculosis (TB) affects a third of mankind and causes 1.7 million fatalities annually [1]. The spread of TB has been facilitated in recent decades due to the susceptibility of HIV-infected individuals to *Mycobacterium tuberculosis*, the etiological agent of TB [2]. The problem has been compounded by the emergence of multi- and extensively-drug resistant *M. tuberculosis* strains [2]. Typically, the cell walls of the genus *Mycobacterium* contain mycolic acids (m), arabinogalactan (AG) and peptidoglycan (P), which are covalently linked to each other to form the mycolyl-arabinogalactan-peptidoglycan (mAGP) complex [3]. A particular group of specialized glycophospholipids, phosphatidyl-*myo*-inositol (PI) mannosides (PIMs) and lipoglycans, lipomannan (LM) and lipoarabinomannan (LAM), are found in the outer leaflet of mAGP [4]. LM and LAM, which are based on a core PIM unit, possess an elongated α(1→6) linear, α(1→2) branched mannan, of approximately 30 mannopyranose (Man*p*) residues, and linked to its terminus to a branched D-arabinan domain of approximately 70 arabinofuranose (Ara*f*) residues, assembled through several distinct structural motifs [4]–[6]. In mycobacteria, the large arabinan domain is capped to various degrees with short α(1→2) Man*p* chains in the case of *M. tuberculosis* [7], whereas in *M. smegmatis*, caps of inositol phosphate are present and termed PILAM [4], and *M. chelonae* possess non-capped LAM [8]. Both ManLAM and PILAM exhibit a broad range of immunomodulatory activities. For example ManLAM, which is predominantly found in the slow-growing pathogenic mycobacteria, inhibits a number of immune system effector functions, including interferon-γ-mediated activation of macrophages [4]. ManLAM also inhibits the production of the pro-inflammatory cytokines interleukin-12 [4] and tumor necrosis factor-α [9]. PILAM, which is characteristic of the fast-growing saprophytic mycobacteria, can induce a pro-inflammatory response in a Toll-like 2 receptor-dependent manner [10].

The current model of lipoglycan biosynthesis is supported by biochemical and genetic studies, and follows a linear pathway from PI→PIM_2_→LM→LAM [4]. PimA (Rv2610c) and PimB’ (Rv2188), are α-mannopyranosyltransferases, belonging to the GT-B superfamily and utilize GDP-mannose [11], [12], adding Man*p* residues at positions *O*-2 and *O*-6 of PI, respectively to produce PIM_2_, that can be subsequently acylated by Rv2611c, at the 6 position of the Man*p* residue to generate Ac_1_/Ac_2_PIM_2_ [13]. RvD2-ORF1 from *M. tuberculosis* CDC1551, designated as PimC and an unknown glycosyltransferase (PimD), catalyzes the addition of a Man*p* residue from GDP-mannose to 6-OH of mannose at the non-reducing end of Ac_1_/Ac_2_PIM_2_ to generate Ac_1_/Ac_2_PIM_3_ [14] and Ac_1_/Ac_2_PIM_4_, respectively. At this key junction point in the biosynthetic pathway, polyprenylphosphate-based mannose (PPM) donors are employed by the GT-C superfamily for elongation and branching of Ac_1_/Ac_2_PIM_4_ leading to Ac_1_/Ac_2_PIM_6_, LM and LAM [15].

The pathway splits into two branches from Ac_1_/Ac_2_PIM_4_: one leads to the formation of Ac_1_/Ac_2_PIM_6,_ whilst the other leads to the formation of LM and LAM. In the first branch, α(1→2) Man*p* is added to Ac_1_/Ac_2_PIM_4_ to form Ac_1_/Ac_2_PIM_6_ by two consecutive mannose additions catalyzed by PimE (Rv1159), a PPM-dependent α(1→2)-mannopyranosyltransferase and a second additional uncharacterized putative glycosyltransferase of the GT-C superfamily [16]_._ In the second branch, Ac_1_/Ac_2_PIM_4_ is hypermannosylated by glycosyltransferases of the GT-C superfamily to generate LM. Recent studies have established MptB (Rv1459c) and MptA (Rv2174) to be involved in the synthesis of the mannan backbone, where MptB catalyzes the synthesis of the proximal end through the addition of 12–15 Man*p* residues to the backbone and MptA synthesizes the distal end of the α(1→6) mannan core of LM [17]–[19]. The α(1→6)-mannan core, synthesized by MptB and MptA, is further branched by MptC (Rv2181) which adds α(1→2)-Man*p* residues to the side chains of LM [20]. The transition from LM to LAM is catalyzed by EmbC (Rv3793) and involves the utilization of LM through initial priming with Ara*f* units from the sugar donor decaprenylphosphate arabinan (DPA), by an unknown GT-C glycosyltransferase [21]. EmbC is then responsible for extension of the primed LM through the addition of 12–16 α(1→5)-Ara*f* residues [22]. The addition of branch points is similar to AG, catalyzed by AftC [23]. The arabinan domain as found for AG synthesis is likely to be terminated by AftB (Rv3805c) [24]. The homologue of Rv1635c in *M. tuberculosis* CDC1551 has been shown to be involved in Man-LAM capping [6]. This enzyme was also shown to be PPM-dependent and is now termed as CapA, which adds the first Man*p* residue onto the non-reducing arabinan termini of LAM [25]. In *M. tuberculosis*, MptC (Rv2181) a PPM-dependent α(1→2) mannosyltransferase has dual functionality in branching LM, and also for producing ManLAM that is fully functional *via* the capping of LAM with α(1→2)-Man*p* residues at the non-reducing end of LAM [4].

**Figure 1:**
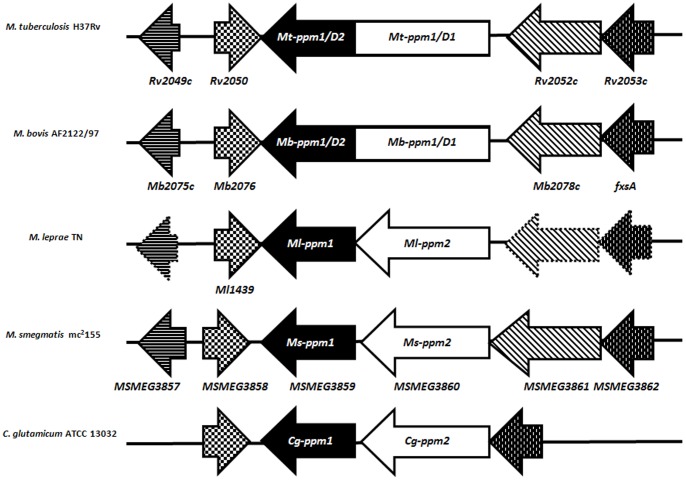
Genomic organization of *ppm1* region in different mycobacteria and in *Corynebacterium glutamicum*. Homologous genes are indicated by similar arrows and pseudogenes in *M. leprae* are indicated by arrows with dotted borders. In *M. tuberculosis* and *M. bovis*, the *ppm1*-encoded protein consist of two domains fused together, while these two domains are encoded by two distinct ORFs in *M. leprae*, *M. smegmatis* and *C. glutamicum*.

*In vitro* studies have shown that the key PPM sugar donor for the GT-C glycosyltransferases involved in LM/LAM biosynthesis is generated by Mt-Ppm1 (Rv2051c), a DPM-like synthase that uses GDP-Man and C_50_/C_35_-polyprenol phosphates as substrates [26]. Previous studies have identified subtle variations in the organization of the *ppm1* locus in mycobacteria [26]. The well characterized *Mt-ppm1* of *M. tuberculosis* encodes a large polypeptide consisting of two domains, *Mt-ppm1/D1*, which is membrane-anchored *via* six transmembrane segments, and *Mt-ppm1/D2* which is sufficient for DPM synthase activity [26], [27]. However, in *Mycobacterium smegmatis*, these two domains are encoded by two distinct ORFs *MSMEG3860* (Domain 1, *Ms-ppm2*) and *MSMEG3859* (Domain 2, *Ms-ppm1*) arranged in an operon (Figure 1), an arrangement also found in *Mycobacterium leprae*, *Mycobacterium avium* and the related *Corynebacterium glutamicum* [26], [28]. Studies using bacterial two-hybrid systems have shown that MSMEG3859 and MSMEG3860 interact with each other [27]; while MSMEG3859 was sufficient for PPM synthase activity, this interaction with MSMEG3860 stabilized the synthase [26], [27]. Similarly, in *M. tuberculosis*, a recombinant protein consisting only of the C-terminal domain (*Mt-Ppm1/D2*) was sufficient for generation of PPM activity [26]. However, whilst *in vitro* data has shown that Mt-Ppm1 (and MSMEG3859) has PPM synthase activity, there have been no genetic studies that demonstrate that Mt-Ppm1 is the sole PPM generating enzyme encoded by the *M. tuberculosis* genome. If this was the case, in view of the critical role of PPM for biosynthesis of LM/LAM, *Mt-ppm1* would be expected to be an essential gene and thus genetic studies would be possible only via the generation of a conditional knockout strain. The need to study the *in vivo* role of *ppm1* by generation of a conditional mutant strain was further necessitated by conflicting reports that another membrane-associated glycosyltransferase, Rv3779 functions as a PPM synthase in *M. tuberculosis* [29], and as a glycosyltransferase that uses polyprenyl-P-D-GalNAc as a D-GalN*p* (or D-GalNAc) donor for transfer to 3,5-branched D-Ara*f* residues of AG [30]. Additionally, the latter report also describes *Rv3631* (*ppgS*) and *Rv3632* as genes encoding a polyprenyl-P-D-GalN synthase and a small integral membrane protein respectively, analogous to Mt-Ppm1/D2 and Mt-Ppm1/D1. While PpgS is a GT-2 family glycosyltransferase involved in the generation of polyprenyl-phospho-*N*-acetylgalactosamine (polyprenyl-P-GalNAc) from polyprenyl-P and UDP-GalNAc, it could be envisaged as a secondary, potential PPM synthase.

In an effort to first confirm the *in vivo* role of Mt-Ppm1/D2 in LM/LAM biosynthesis we aimed to test the essentiality of PPM glycosyltransferase activity in *M. smegmatis* by using CESTET, a genetic tool for testing gene essentiality in *M. smegmatis*. As *M. smegmatis* does not encode homologues of *ppgS* or *Rv3779*, it also provided us with a valuable surrogate to probe the *in vivo* role and potential essentiality of *MSMEG3859* in the absence of any potential functional redundancy caused by an alternative PPM synthase. Additionally, mutant or conditional mutant strains of *M. smegmatis* could subsequently be used as a host strain to test functional complementation of PPM synthase activity using recombinant *ppgS* or *Rv3779*.

## Materials and Methods

### Construction of Recombinant Plasmids

For generating an integrative vector containing *MSMEG3859*, the ORF was amplified from *M. smegmatis* mc^2^155 [31] genomic DNA using the primers F3859 (5′-TCGGAATTCATGAGCGTCCCCGGTGAACG-3′) and R3859 (5′-GCTATCGATTCAGCGGACCACGCCCCTGG-3′), cloned downstream of the tetracycline promoter in the integrative vector pTIC6a vector (gift from A. Baughn and W.R. Jacobs Jr., Albert Einstein College of Medicine, NY) and named pTIC6a-*MSMEG3859*. For subsequent complementation/rescue experiments, the plasmid pMV261Apra, a derivative of pMV261 [32], was used for cloning various GTFs. *MSMEG3859* was amplified using primer pairs F3859A (5′-GCGGTCAGCTGATGAGCGTCCCCG-3′) and R3859 (5′-GCTATCGATTC AGCGGACCACGCCCCTGG-3′); the C-terminal domain of *Rv2051c* (*Mt-ppm1/D2*) using primer pairs F2051C (5′-GCTGCTGGCCAACCACCGGCCAGC-3′) and R2051C (5′-TCGGAATTCACCACCGGCCAGCCG-3′); *Rv3779* using F3779 (5′-GATGGCCAGTGGGCCTGTGGTTCG-3′) and R3779 (5′-ATAAGCTTCC TAGGAGTGTGTTGC-3′); *Rv3631* (*ppgS*) using F3631 (5′-GCTCGTGGCCAA TGGCCTCGAAAA-3′) and R3631 (5′-TCGACATCGATTCATCGTGGCATC-3′). The-PCR amplified DNA fragments of *MSMEG3859*, *Mt-ppm1/D2*, *Rv3779* and *ppgS* were cloned into pMV261Apra using primer-incorporated restriction sites and the resultant plasmids were named pAKR-*MSMEG3859*, pAKR-*Mt-ppm1/D2*, pAKR-*Rv3779* and pAKR-*ppgS*, respectively.

### Generation of the ΔMsPpm Conditional Mutant

The *M. smegmatis* conditional mutant ΔMsPpm was generated using CESTET [33]. Briefly, a merodiploid was first generated by introducing pTIC6a-*MSMEG3859* by electroporation into *M. smegmatis* mc^2^155 [33]. The merodiploid strain mc^2^155::pTIC6a-*MSMEG3859* was then subjected to specialized transduction as previously described [34] using a temperature-sensitive, recombinant phage phΔMsPpm designed to replace *MSMEG3859-MSMEG3860* with a hygromycin resistance marker. Transductants were selected at the non-permissive temperature of 37°C on selective plates containing 25 µg/ml kanamycin, 100 µg/ml hygromycin B and 50 ng/ml anhydrotetracycline (ATc). After confirmation of gene replacement by Southern blot, one such transductant was named ΔMsPpm and was selected for further analysis.

### Conditional Depletion of ΔMsPpm Conditional Mutant

The ΔMsPpm conditional mutant was grown in Tryptic Soy Broth (TSB; Difco) containing 0.05% Tween 80, 25 µg/ml kanamycin, 100 µg/ml hygromycin B and 50 ng/ml ATc and subsequently passaged twice in medium without ATc. To visualize the effects of the conditional depletion of *MSMEG3859* in ΔMsPpm on lipids and lipoglycans, the strains were grown to OD 0.8, labelled with 10 µCi/ml glucose D-[^14^C(U)] (specific activity 250–360 mCi (9.25–13.3 GBq)/mmol; Perkin Elmer) and incubated at 37°C for 4 hours.

### Extraction of Polar Lipids

Polar lipids and apolar lipids were extracted as described previously [35]. Briefly, cells from a 10 ml volume culture were washed once with 2 ml phosphate buffer saline (PBS) and treated with 2 ml CH_3_OH:0.3% NaCl_(aq)_ (100∶10, v/v) and 2 ml petroleum ether for 30 min. The suspension was centrifuged and the upper layer containing apolar lipids was separated. An additional 2 ml of petroleum ether was added, mixed and centrifuged as described above and the two upper apolar lipid fractions were combined and dried. For polar lipids, 2.3 ml of CHCl_3_:CH_3_OH:0.3% NaCl_(aq)_ (90∶100∶30, v/v/v) was added to the cell pellet and mixed for 1 h. This mixture was centrifuged and the supernatant was separated. The remaining cell pellet was mixed with 750 µl of CHCl_3_:CH_3_OH:0.3% NaCl_(aq)_ (50∶100∶40, v/v/v) for 30 min, centrifuged and the supernatant was combined to the previous fraction. After repeating this step, 1.3 ml of CHCl_3_ and 1.3 ml of 0.3% NaCl_(aq)_ was mixed with the pooled supernatant, centrifuged, and the lower layer containing the polar lipids was recovered and dried. The polar lipid extracts were dried and resuspended in CHCl_3_:CH_3_OH (2 : 1, v/v), and incorporation of glucose D-[^14^C(U)] was quantified by liquid scintillation counting using 5 % of the lipid fractions in 5 ml EcoScint A (National Diagnostics). Equal counts of polar lipid extracts (50 000 cpm) were applied to Silica Gel 60 F_254_ (Merck 5554) aluminium-backed TLC plates and developed using solvent system E for polar lipids: CHCl_3_:CH_3_OH:H_2_O (60∶30∶6, v/v/v) in the first direction and CHCl_3_:CH_3_CO_2_H:CH_3_OH:H_2_O (40∶25∶3∶6, v/v/v/v) in the second direction. Polar lipids were visualized by 48 h exposure on x-ray films by autoradiography (Kodak Biomax MR film).

### Extraction and Purification of Lipoglycans

Lipoglycans were extracted as described previously [36]. Briefly, dried cells from a 10 ml volume culture were resuspended in water and refluxed five times with equal volume of 50% C_2_H_5_OH at 85°C, for 6 h intervals, followed by centrifugation and recovery of the supernatant. The combined supernatants were dried and subjected to hot phenol-H_2_O treatment at 65°C. The aqueous phase containing the crude lipoglycan fraction was dialyzed against water, dried and the incorporation of glucose D-[^14^C(U)] was quantified by liquid scintillation counting using 5 % of the lipoglycan fractions in 5 ml EcoScint A (National Diagnostics). Equal counts (50 000 cpm) were loaded on a 15% SDS-PAGE gel and separated by electrophoresis. Lipoglycans were visualized by 48 h exposure on x-ray films by autoradiography (Kodak Biomax MR film).

### PPM Synthase Assay

Membranes were prepared as described previously [37]. Briefly, cells were grown to mid-log phase, harvested, washed with PBS and stored at –20°C. Cells were washed and resuspended in buffer A containing 50 mM MOPS (adjusted to pH 7.9 with KOH), 5 mM β-mercaptoethanol and 10 mM MgCl_2(aq)_ at 4°C and subjected to sonication for a total time of 10 min using 60 s pulses and 90 s cooling intervals. The preparations were centrifuged at 27000×*g* for 25 min at 4°C and the membranes were obtained by further centrifugation of the supernatant at 100,000×*g* for 1 h at 4°C. Membranes were resuspended in 1 ml of buffer A and concentration was determined using the BCA Protein Assay Reagent Kit (Thermo Scientific). Reaction mixtures for assessing [^14^C]Man incorporation consisted of 6.25 µCi GDP[Man-^14^C(U)] (262 mCi/mmol; Perkin Elmer), 100 µM ATP, 10 mM MgCl_2(aq)_, 100 µM dithiothreitol, 20 mM NaF_(aq)_ and membrane preparations corresponding to 50–400 µg protein in a final volume of 100 µl. Decaprenyl monophosphate was added to the reaction mixtures at a final concentration of 125 µM. The reaction mixtures were then incubated at 37°C for 30 min. The enzymic reactions were terminated by the addition CHCl_3_/CH_3_OH/0.8 M NaOH_(aq)_ (10∶10∶3 by vol.) (6 ml/100 µl) followed by further incubation at 55°C for 20 min. The mixtures were then allowed to cool; 2.625 ml of CHCl_3_ and 1.125 ml of water were added. The mixture was vortexed and centrifuged and the upper aqueous phase discarded. The organic phase was washed three times with 2 ml of CHCl_3_:CH_3_OH:H_2_O (3∶47∶48 by vol.), dried to yield an organic fraction containing PPMs. These were dried in a scintillation vial before scintillation counting using 5 ml of EcoScint A (National Diagnostics) [26].

## Results and Discussion

### Essentiality of MSMEG3859 in M. smegmatis

**Figure 2:**
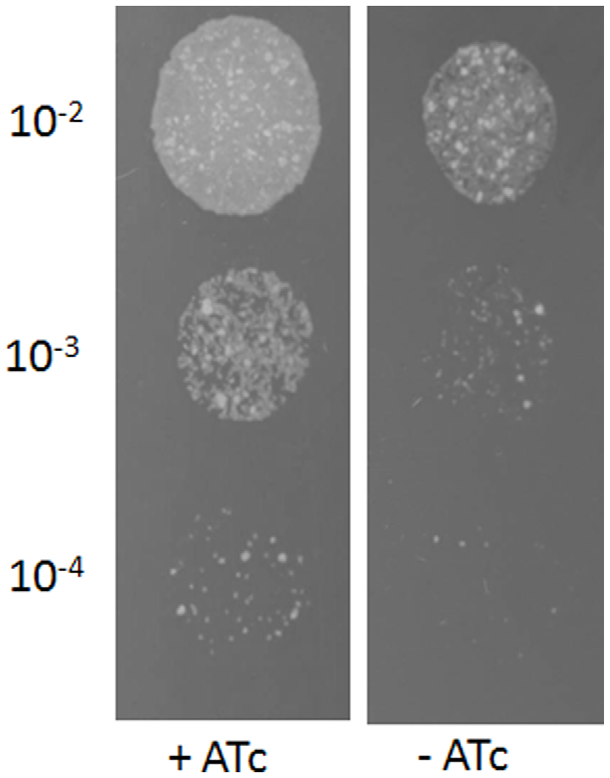
Essentiality of *MSMEG3859* in *M. smegmatis* mc^2^155. Growth of the ΔMsPpm conditional mutant on Tryptic Soy Agar with or without the inducer anhydrotetracycline (ATc). Ten microliters of 10-fold serial dilutions of cultures were spotted on the agar plates and incubated for 3 days at 37°C.

Due to its role in the biosynthesis of LM/LAM, *Mt-ppm1* was considered to be an essential gene. We constructed a knockout phage phΔMsPpm designed to replace *MSMEG3859*-*MSMEG3860* in *M. smegmatis* with a hygromycin resistance cassette; we were however unable to generate a null mutant due to the failure to yield any transductants. In contrast, we were able to generate a *MSMEG3859*-*MSMEG3860* null mutant by transducing a merodiploid strain containing a second, inducible copy of *MSMEG3859*-*MSMEG3860* by CESTET (Conditional Expression Specialized Transduction Essentiality Test) [33]; suggesting that one or both genes were essential in *M. smegmatis*. As MSMEG3859 was shown to be sufficient for the enzymatic generation of PPM *in vitro*, we reasoned that the PPM synthase-encoding *MSMEG3859*, but not the membrane segment-encoding *MSMEG3860*, was an essential gene. To address this, we used CESTET again to test the essentiality of *MSMEG3859* in *M. smegmatis*. First, a merodiploid strain was constructed by introducing a second copy of *MSMEG3859* cloned in pTIC6a, an integrative plasmid driving expression *via* a tetracycline inducible promoter [38]. Expression of this recombinant copy of *MSMEG3859* can be induced by adding anhydrotetracycline (ATc) to the growth medium. Following transduction with knockout phage phΔMsPpm, we were able to generate knockout mutants only when transductants were selected on plates containing hygromycin and ATc suggesting that *MSMEG3859* was essential in *M. smegmatis*. One such conditional mutant, designated ΔMsPpm was used for further analysis. Subsequent passages of the ΔMsPpm mutant in medium without the inducer ATc resulted in loss of viability indicating that expression of the pTIC6a-driven copy of *MSMEG3859* was required for cell growth, confirming the essentiality of *MSMEG3859* (Figure 2).

The ability to generate an *MSMEG3859-MSMEG3860* double mutant in a *MSMEG3859* merodiploid strain indicated that while the PPM synthase-encoding *MSMEG3859* was essential, *MSMEG3860* (which encodes a six transmembrane section-containing membrane-anchored protein) was not essential. This correlated with earlier reports that *MSMEG3859* was sufficient for PPM synthase activity [26], [27].

### Loss of *MSMEG3859* Results in Alteration of PIMs and Cessation of LM Biosynthesis

**Figure 3:**
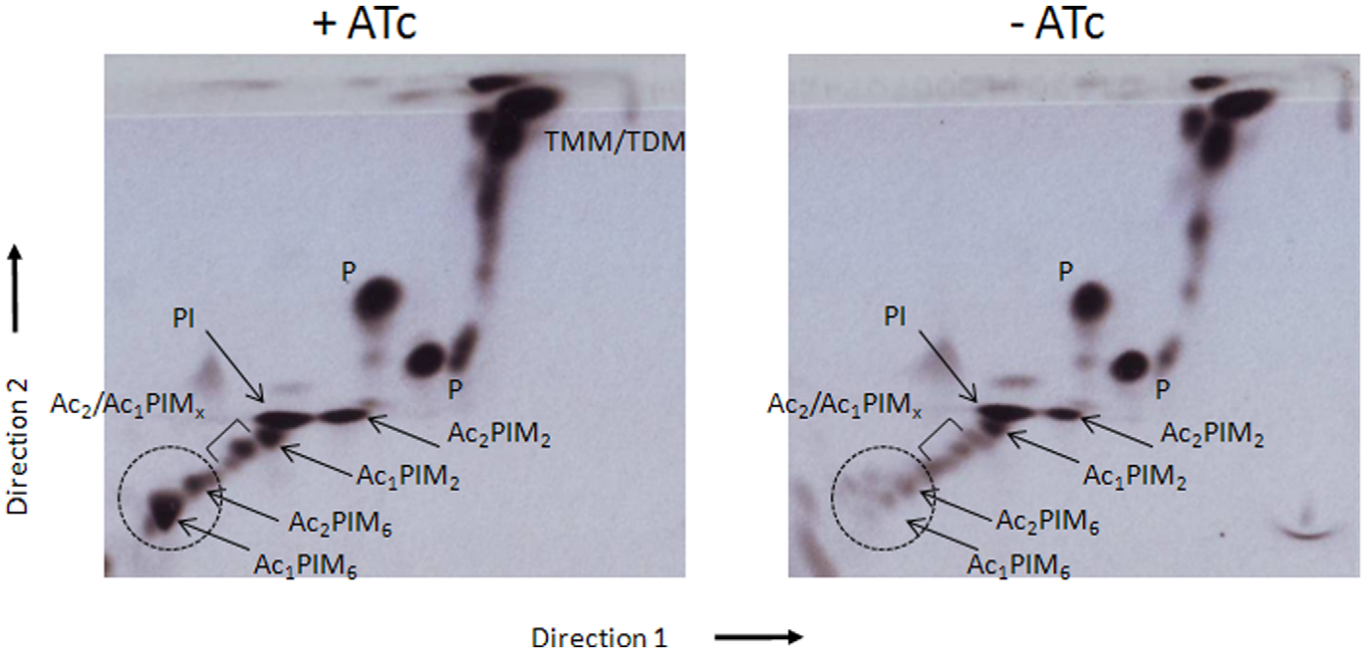
2D-TLC analysis of [^14^C]-labeled polar lipids from the ΔMsPpm conditional mutant. Cultures were grown and labelled in Tryptic Soy Broth in the presence or the absence of anhydrotetracycline (ATc). Equal counts of polar lipid extracts (50 000 cpm) were applied to Silica Gel 60 F_254_ (Merck 5554) aluminium-backed TLC plates and developed using solvent system E for polar lipids: CHCl_3_/CH_3_OH/H_2_O (60∶30∶6, v/v/v) in the first direction and CHCl_3_:CH_3_OOH: CH_3_OH:H_2_O (40∶25∶3∶6, v/v/v/v) in the second direction. Polar lipids were visualized by 48 h exposure on x-ray films by autoradiography (Kodak Biomax MR film). PIM intermediates are shown by arrows (x = 3–5) and the dotted circle indicates the position of the higher PIMs on the TLC plates.

**Figure 4:**
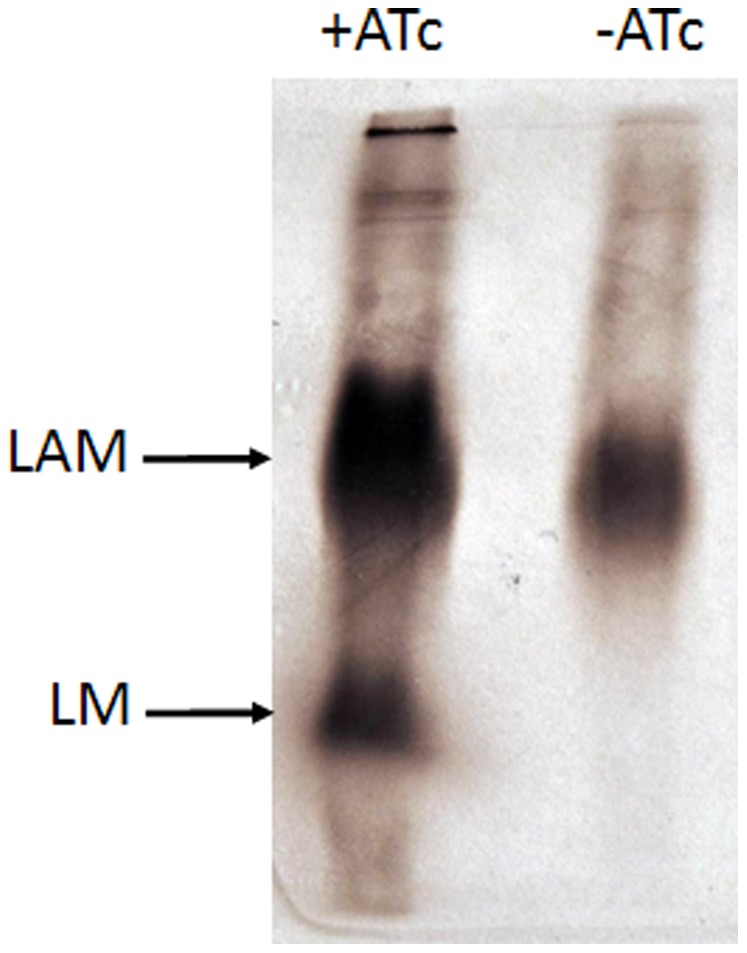
Lipoglycan profile of the ΔMsPpm conditional mutant. [^14^C]-labelled lipoglycan fractions were separated on 15% SDS-PAGE gel from cultures of the ΔMsPpm conditional mutant with or without anhydrotetracycline (ATc). Equal counts (50 000 cpm) were loaded on a 15% SDS-PAGE. Lipoglycans were visualized by 48 h exposure on x-ray films by autoradiography (Kodak Biomax MR film).

As Ac_1_/Ac_2_PIM_4_ is at the branch point for the biosynthesis of higher PIMs (Ac_1_/Ac_2_PIM_6_) and LM/LAM biosynthesis, the conditional ΔMsPpm mutant could be used to determine whether loss of *MSMEG3859*-encoded PPM activity affected the biosynthesis of these molecules. The mutant was grown for 36 hours in media in the presence, or absence, of ATc labelled with [^14^C]-glucose and subjected to lipid extractions. Cultures grown in ATc-containing media showed all PIM intermediates present. In contrast, cultures of the conditional mutant grown in the absence of ATc showed increasing amounts of PI accompanied by decreasing levels of Ac_1_/Ac_2_PIM_6_ (Figure 3). The loss of Ac_1_/Ac_2_PIM_6_ and accumulation of PIM_x_ precursors suggested that *MSMEG3859* is required for the synthesis of higher order PIMs, particularly Ac_1_/Ac_2_PIM_6_. Conditional depletion of PPM synthase activity in the ΔMsPpm mutant should also affect the biosynthesis of lipoglycans and cultures grown in the absence of ATc did show diminished levels of [^14^C]LM (Figure 4). However, we did not see major differences in [^14^C]LAM levels. The loss of [^14^C]LM but presence of [^14^C]LAM in the depleted cultures could be explained by the residual LM molecules in the depleted cells being extended to form [^14^C]LAM from endogenous PPM generated prior to conditional depletion. Thus, the observed LAM in the depleted cells was likely from residual LM, rather than newly synthesised LM, as observed through dual [^14^C]/[^3^H] dual labelling experiments by Besra et al. [37]. These data suggest that *MSMEG3859*, which encodes the equivalent of *M. tuberculosis* Ppm1/D2, is solely responsible for the PPM synthase activity required for the generation of higher order PIMs and LM/LAM.

### Effects of Loss of MSMEG3859 on Membrane-associated PPM Synthase Activity

**Figure 5:**
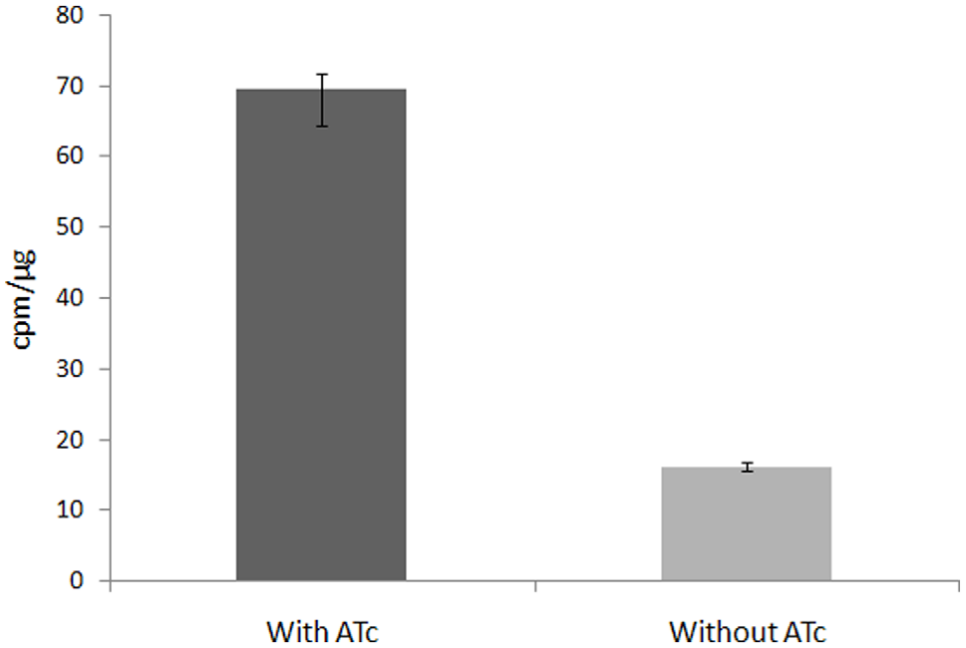
PPM synthase activity in membranes of the ΔMsPpm conditional mutant. Graph shows the transfer of [^14^C]-mannose from GDP[Man-^14^C(U)] (262 mCi/mmol; Perkin Elmer) to polyprenol-phosphate in membrane extracts prepared from cultures grown with or without anhydrotetracycline (ATc).

Membrane preparations from cultures of the ΔMsPpm mutant, grown in the presence and in the absence of ATc, were used to assay membrane-associated PPM synthase activity. A PPM synthase assay was used to analyse PPM activity in the membranes using GDP-[^14^C] Man and polyprenol phosphate as substrate. This assay was used to assess the effects of depletion of MSMEG3859 function on the ability of the membrane preparations to catalyse the formation of PPM (C_50_-P-Man) donors *via* the incorporation of radioactive mannose into polyprenol substrate through pooled organic extracts containing PPMs [26]. While membrane preparations from cultures grown in the presence of ATc were able to catalyse incorporation of [^14^C] Man into polyprenols, those from cultures grown in the absence of ATc displayed poor PPM synthase activity (Figure 5). This co-relation between depleted MSMEG3859 function and low PPM synthase activity confirmed that MSMEG3859 was the key synthase required to catalyze the production of PPM donors in *M. smegmatis*.

Together, the data obtained from the depletion experiments with the conditional mutant highlight the potential of the *MSMEG3859*-encoded PPM synthase as a potential drug target that affects not only viability, but also the biosynthesis of a immunomodulatory mycobacterial lipoglycan.

### Potential Ability of the Alternative *M. tuberculosis* PPM Synthases to Rescue Viability and Restore Wild-type Phenotype in the ΔMsPpm Mutant

**Figure 6:**
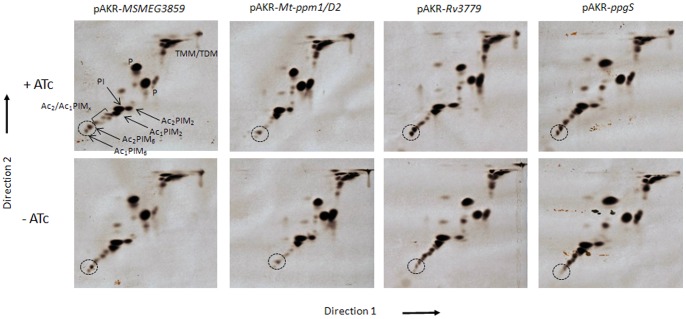
Complementation of the ΔMsPpm conditional mutant. 2D-TLC analysis of [^14^C]-labelled polar lipids from the ΔMsPpm conditional mutant complemented with (A) pAKR- *MSMEG3859* (B) pAKR-*Mt-ppm1/D2* (C) pAKR-*Rv3779* (D) pAKR-*ppgS*. Equal counts of polar lipid extracts (50 000 cpm) were applied to Silica Gel 60 F_254_ (Merck 5554) aluminium-backed TLC plates and developed using solvent system E for polar lipids: CHCl_3_/CH_3_OH/H_2_O (60∶30∶6, v/v/v) in the first direction and CHCl_3_:CH_3_OOH: CH_3_OH:H_2_O (40∶25∶3∶6, v/v/v/v) in the second direction. Polar lipids were visualized by 48 h exposure on x-ray films by autoradiography (Kodak Biomax MR film). PIM intermediates are shown by arrows (x = 3–5) and the dotted circle indicates the position of the higher PIMs on the TLC plates.

In contrast to *M. smegmatis*, *M. tuberculosis* encodes two other membrane-associated glycosyltransferases, Rv3779 and Rv3631 (PpgS), which have been suggested to have putative roles as PPM synthases in *M. tuberculosis* [30]. As mentioned above, homologues of neither are not present in *M. smegmatis*. The ΔMsPpm conditional mutant thus offered us an opportunity to assess the possible roles of Rv3779 and PpgS as alternative PPM synthases. Each gene could be functionally analyzed *in vivo* by testing the ability of the ΔMsPpm conditional mutant transformed with a plasmid-borne copy of either *Rv3779* or *ppgS* to rescue Ac_1_/Ac_2_PIM_6_ biosynthesis when cultured in medium devoid of ATc. As expected, Ac_1_/Ac_2_PIM_6_ biosynthesis was not affected in non-ATc cultures of ΔMsPpm containing plasmid clones of either *MSMEG3859* (Figure 6). We then tested the ability of the *M. tuberculosis* equivalent, *Mt-ppm1/D2* to rescue the loss of *MSMEG3859* function in the same manner and found Ac_1_/Ac_2_PIM_6_ levels unaffected in the recombinant strains (Figure 6) indicating that *Mt-ppm1/D2* was functional in *M. smegmatis*. In contrast, however, *Rv3779* was unable to rescue Ac_1_/Ac_2_PIM_6_ biosynthesis in the conditional mutant when grown in the absence of ATc, leading to a loss of Ac_1_/Ac_2_PIM_6_ and a noticeable accumulation of intermediary Ac_1_/Ac_2_PIM_x_s (Figure 6). Thus while Rv3779 was shown to have PPM synthase activity *in vitro* [29], it surprisingly failed to substitute for loss of MSMEG3859 in the conditional ΔMsPpm mutant. Thus, in contrast to its *in vitro* activity, it is unlikely that Rv3779 functions *in vivo* as a PPM synthase. Instead, its predominant role in *M. tuberculosis* seems to be the utilisation of polyprenyl-P-D-GalNAc as a donor for the biosynthesis of galactosamine-modified AG [30].

As mentioned above, while PpgS is involved in the generation of polyprenyl-phospho-*N*-acetylgalactosamine (polyprenyl-P-GalNAc) from polyprenyl-P and UDP-GalNAc, it could be envisaged as a secondary, potential PPM synthase. Its syntenic association with the small integral membrane protein Rv3662 is analogous to MSMEG3859-MSMEG3860, and Mt-Ppm1/D2-Mt-Ppm1/D1. However, *ppgS* also failed to compensate for the loss of MSMEG3859 in the conditional mutant (Figure 6), and thus is unlikely to function as a PPM synthase *in vivo*.

Together, these results indicate that *ppm1* is the gene encoding the sole PPM synthase in *M. tuberculosis* capable of generating the mannose donor for subsequent higher order PIMs and LM/LAM biosynthesis.

## References

[1] WHO (2010) WHO report 2010: Global tuberculosis control. World Health Organization, Geneva.

[2] WHO (2010) The WHO: multidrug and extensively drug resistant TB (M/XDR-TB): 2010 global report on surveillance and response.

[3] Jankute M, Grover S, Rana AK, Besra GS (2012) Arabinogalactan and lipoarabinomannan biosynthesis: structure, biogenesis and their potential as drug targets. Future Microbiol 7: 129–147. 22191451 10.2217/fmb.11.123

[4] Mishra AK, Driessen NN, Appelmelk BJ, Besra GS (2011) Lipoarabinomannan and related glycoconjugates: structure, biogenesis and role in *Mycobacterium tuberculosis* physiology and host-pathogen interaction. FEMS Microbiol Rev 35: 1126–1157. 21521247 10.1111/j.1574-6976.2011.00276.x PMC3229680

[5] Birch HL, Alderwick LJ, Appelmelk BJ, Maaskant J, Bhatt A, et al (2010) A truncated lipoglycan from mycobacteria with altered immunological properties. Proc Natl Acad Sci USA 107: 2634–2639. 20133807 10.1073/pnas.0915082107 PMC2823879

[6] Dinadayala P, Kaur D, Berg S, Amin AG, Vissa VD, et al (2006) Genetic basis for the synthesis of the immunomodulatory mannose caps of lipoarabinomannan in *Mycobacterium tuberculosis* . J Biol Chem 281: 20027–20035. 16704981 10.1074/jbc.M603395200

[7] Chatterjee D, Khoo KH, McNeil MR, Dell A, Morris HR, et al (1993) Structural definition of the non-reducing termini of mannose-capped LAM from *Mycobacterium tuberculosis* through selective enzymatic degradation and fast atom bombardment-mass spectrometry. Glycobiology 3: 497–506. 8286863 10.1093/glycob/3.5.497

[8] Guerardel Y, Maes E, Elass E, Leroy Y, Timmerman P, et al (2002) Structural study of lipomannan and lipoarabinomannan from *Mycobacterium chelonae*. Presence of unusual components with alpha 1,3-mannopyranose side chains. J Biol Chem 277: 30635–30648. 12063260 10.1074/jbc.M204398200

[9] Mahon RN, Rojas RE, Fulton SA, Franko JL, Harding CV, et al (2009) *Mycobacterium tuberculosis* cell wall glycolipids directly inhibit CD4+ T-cell activation by interfering with proximal T-cell-receptor signaling. Infect Immun 77: 4574–4583. 19651854 10.1128/IAI.00222-09 PMC2747961

[10] Vignal C, Guérardel Y, Kremer L, Masson M, Legrand D, et al (2003) Lipomannans, but not lipoarabinomannans, purified from *Mycobacterium chelonae* and *Mycobacterium kansasii* induce TNF-alpha and IL-8 secretion by a CD14-toll-like receptor 2-dependent mechanism. J Immunol 171: 2014–2023. 12902506 10.4049/jimmunol.171.4.2014

[11] Guerin ME, Kordulakova J, Schaeffer F, Svetlikova Z, Buschiazzo A, et al (2007) Molecular recognition and interfacial catalysis by the essential phosphatidylinositol mannosyltransferase PimA from mycobacteria. The J Biol Chem 282: 20705–20714. 17510062 10.1074/jbc.M702087200

[12] Mishra AK, Klein C, Gurcha SS, Alderwick LJ, Babu P, et al (2008) Structural characterization and functional properties of a novel lipomannan variant isolated from a *Corynebacterium glutamicum* pimB’ mutant. Antonie van Leeuwenhoek 94: 277–287. 18421567 10.1007/s10482-008-9243-1 PMC2480597

[13] Korduláková J, Gilleron M, Puzo G, Brennan PJ, Gicquel B, et al (2003) Identification of the required acyltransferase step in the biosynthesis of the phosphatidylinositol mannosides of mycobacterium species. J Biol Chem 278: 36285–36295. 12851411 10.1074/jbc.M303639200

[14] Kremer L, Gurcha SS, Bifani P, Hitchen PG, Baulard A, et al (2002) Characterization of a putative alpha-mannosyltransferase involved in phosphatidylinositol trimannoside biosynthesis in *Mycobacterium tuberculosis* . Biochem J 363: 437–447. 11964144 10.1042/0264-6021:3630437 PMC1222496

[15] Guerin ME, Korduláková J, Alzari PM, Brennan PJ, Jackson M (2010) Molecular basis of phosphatidyl-myo-inositol mannoside biosynthesis and regulation in mycobacteria. J Biol Chem 285: 33577–33583. 20801880 10.1074/jbc.R110.168328 PMC2962455

[16] Berg S, Kaur D, Jackson M, Brennan PJ (2007) The glycosyltransferases of *Mycobacterium tuberculosis* - roles in the synthesis of arabinogalactan, lipoarabinomannan, and other glycoconjugates. Glycobiology 17: 35–56R. 17261566 10.1093/glycob/cwm010

[17] Mishra AK, Alderwick LJ, Rittmann D, Wang C, Bhatt A, et al (2008) Identification of a novel alpha(1–>6) mannopyranosyltransferase MptB from *Corynebacterium glutamicum* by deletion of a conserved gene, NCgl1505, affords a lipomannan- and lipoarabinomannan-deficient mutant. Mol Microbiol 68: 1595–1613. 18452585 10.1111/j.1365-2958.2008.06265.x PMC2440535

[18] Kaur D, McNeil MR, Khoo K-H, Chatterjee D, Crick DC, et al (2007) New insights into the biosynthesis of mycobacterial lipomannan arising from deletion of a conserved gene. J Biol Chem 282: 27133–27140. 17606615 10.1074/jbc.M703389200

[19] Mishra AK, Alderwick LJ, Rittmann D, Tatituri RVV, Nigou J, et al (2007) Identification of an alpha(1–>6) mannopyranosyltransferase (MptA), involved in *Corynebacterium glutamicum* lipomanann biosynthesis, and identification of its orthologue in *Mycobacterium tuberculosis* . Mol Microbiol 65: 1503–1517. 17714444 10.1111/j.1365-2958.2007.05884.x PMC2157549

[20] Mishra AK, Krumbach K, Rittmann D, Appelmelk B, Pathak V, et al (2011) Lipoarabinomannan biosynthesis in Corynebacterineae: the interplay of two α(1→2)-mannopyranosyltransferases MptC and MptD in mannan branching. Mol Microbiol 80: 1241–1259. 21435038 10.1111/j.1365-2958.2011.07640.x PMC3123699

[21] Wolucka BA (2008) Biosynthesis of D-arabinose in mycobacteria - a novel bacterial pathway with implications for antimycobacterial therapy. FEBS J 275: 2691–2711. 18422659 10.1111/j.1742-4658.2008.06395.x

[22] Zhang N, Torrelles JB, McNeil MR, Escuyer VE, Khoo K-H, et al (2003) The Emb proteins of mycobacteria direct arabinosylation of lipoarabinomannan and arabinogalactan via an N-terminal recognition region and a C-terminal synthetic region. Mol Microbiol 50: 69–76. 14507364 10.1046/j.1365-2958.2003.03681.x

[23] Birch HL, Alderwick LJ, Bhatt A, Rittmann D, Krumbach K, et al (2008) Biosynthesis of mycobacterial arabinogalactan: identification of a novel alpha(1–>3) arabinofuranosyltransferase. Mol Microbiol 69: 1191–1206. 18627460 10.1111/j.1365-2958.2008.06354.x PMC2610374

[24] Seidel M, Alderwick LJ, Birch HL, Sahm H, Eggeling L, et al (2007) Identification of a novel arabinofuranosyltransferase AftB involved in a terminal step of cell wall arabinan biosynthesis in Corynebacterianeae, such as *Corynebacterium glutamicum* and *Mycobacterium tuberculosis* . J Biol Chem 282: 14729–14740. 17387176 10.1074/jbc.M700271200

[25] Appelmelk BJ, den Dunnen J, Driessen NN, Ummels R, Pak M, et al (2008) The mannose cap of mycobacterial lipoarabinomannan does not dominate the *Mycobacterium*-host interaction. Cell Microbiol 10: 930–944. 18070119 10.1111/j.1462-5822.2007.01097.x

[26] Gurcha SS, Baulard AR, Kremer L, Locht C, Moody DB, et al (2002) Ppm1, a novel polyprenol monophosphomannose synthase from *Mycobacterium tuberculosis* . Biochem J 365: 441–450. 11931640 10.1042/BJ20020107 PMC1222681

[27] Baulard AR, Gurcha SS, Engohang-Ndong J, Gouffi K, Locht C, et al (2003) In vivo interaction between the polyprenol phosphate mannose synthase Ppm1 and the integral membrane protein Ppm2 from *Mycobacterium smegmatis* revealed by a bacterial two-hybrid system. J Biol Chem 278: 2242–2248. 12427759 10.1074/jbc.M207922200

[28] Gibson KJC, Eggeling L, Maughan WN, Krumbach K, Gurcha SS, et al (2003) Disruption of Cg-Ppm1, a polyprenyl monophosphomannose synthase, and the generation of lipoglycan-less mutants in *Corynebacterium glutamicum* . J Biol Chem 278: 40842–40850. 12904287 10.1074/jbc.M307988200

[29] Scherman H, Kaur D, Pham H, Skovierová H, Jackson M, et al (2009) Identification of a polyprenylphosphomannosyl synthase involved in the synthesis of mycobacterial mannosides. J Bacteriol 191: 6769–6772. 19717608 10.1128/JB.00431-09 PMC2795309

[30] Skovierová H, Larrouy-Maumus G, Pham H, Belanová M, Barilone N, et al (2010) Biosynthetic origin of the galactosamine substituent of Arabinogalactan in *Mycobacterium tuberculosis* . J Biol Chem 285: 41348–41355. 21030587 10.1074/jbc.M110.188110 PMC3009860

[31] Snapper SB, Melton RE, Mustafa S, Kieser T, Jacobs WR (1990) Isolation and characterization of efficient plasmid transformation mutants of *Mycobacterium smegmatis* . Mol Microbiol 4: 1911–1919. 2082148 10.1111/j.1365-2958.1990.tb02040.x

[32] Stover CK, de la Cruz VF, Fuerst TR, Burlein JE, Benson LA, et al (1991) New use of BCG for recombinant vaccines. Nature 351: 456–460. 1904554 10.1038/351456a0

[33] Bhatt A, Kremer L, Dai AZ, Sacchettini JC, Jacobs WR (2005) Conditional depletion of KasA, a key enzyme of mycolic acid biosynthesis, leads to mycobacterial cell lysis. J Bacteriol 187: 7596–7606. 16267284 10.1128/JB.187.22.7596-7606.2005 PMC1280301

[34] Bardarov S, Bardarov S Jr, Pavelka MS Jr, Sambandamurthy V, Larsen M, et al (2002) Specialized transduction: an efficient method for generating marked and unmarked targeted gene disruptions in *Mycobacterium tuberculosis*, *M. bovis* BCG and *M. smegmatis* . Microbiology 148: 3007–3017. 12368434 10.1099/00221287-148-10-3007

[35] Dobson G, Minnikin DE, Minnikin SM, Parlett JH, Goodfellow M, et al.. (1985) Systematic analysis of complex mycobacterial lipids. In: Goodfellow M MDE, editor. Chemical Methods in Bacterial Systematics. London: Academic Press. 237–265.

[36] Ludwiczak P, Brando T, Monsarrat B, Puzo G (2001) Structural characterization of *Mycobacterium tuberculosis* lipoarabinomannans by the combination of capillary electrophoresis and matrix-assisted laser desorption/ionization time-of-flight mass spectrometry. Anal Chem 73: 2323–2330. 11393859 10.1021/ac001368h

[37] Besra GS, Morehouse CB, Rittner CM, Waechter CJ, Brennan PJ (1997) Biosynthesis of mycobacterial lipoarabinomannan. J Biol Chem 272: 18460–18466. 9218490 10.1074/jbc.272.29.18460

[38] Ehrt S, Guo XV, Hickey CM, Ryou M, Monteleone M, et al (2005) Controlling gene expression in mycobacteria with anhydrotetracycline and Tet repressor. Nucleic Acids Res 33: e21. 15687379 10.1093/nar/gni013 PMC548372

